# Passive Detection of Ship-Radiated Acoustic Signal Using Coherent Integration of Cross-Power Spectrum with Doppler and Time Delay Compensations

**DOI:** 10.3390/s20061767

**Published:** 2020-03-22

**Authors:** Wei Guo, Shengchun Piao, Junyuan Guo, Yahui Lei, Kashif Iqbal

**Affiliations:** 1 Acoustic Science and Technology Laboratory, Harbin Engineering University, Harbin 150001, China; guowei1030@hrbeu.edu.cn (W.G.); piaoshengchun@hrbeu.edu.cn (S.P.); leiyahui@hrbeu.edu.cn (Y.L.); 2 Key Laboratory of Marine Information Acquisition and Security (Harbin Engineering University), Ministry of Industry and Information Technology, Harbin 150001, China; 3 College of Underwater Acoustic Engineering, Harbin Engineering University, Harbin 150001, China; kashifjamshed607@hrbeu.edu.cn

**Keywords:** passive sonar array, ship-radiated acoustic signal, Doppler, cross-power spectrum, coherent integration

## Abstract

Passive sonar is widely used for target detection, identification and classification based on the target radiated acoustic signal. Under the influence of Doppler, generated by relative motion between the moving target and the sonar array, the received ship-radiated acoustic signals are non-stationary and time-varying, which has a negative effect on target detection and other fields. In order to reduce the influence of Doppler and improve the performance of target detection, a coherent integration method based on cross-power spectrum is proposed in this paper. It can be concluded that the frequency shift and phase change in the cross-power spectrum obtained by each pair of data segments can be corrected with the compensations of time scale (Doppler) factor and time delay. Moreover, the time scale factor and time delay can be estimated from the amplitude and phase of the original cross-power spectrum, respectively. Therefore, coherent integration can be implemented with the compensated cross-power spectra. Simulation and experimental data processing results show that the proposed method can provide sufficient processing gains and effectively extract the discrete spectra for the detection of moving targets.

## 1. Introduction

**Figure 1:**
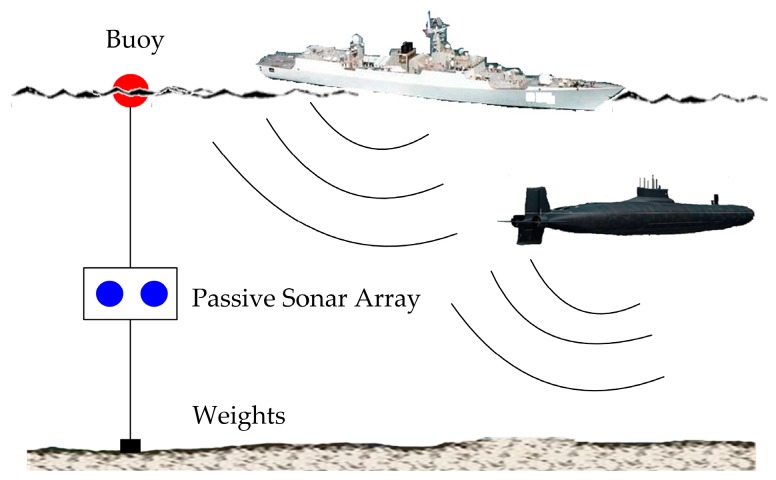
Passive sonar array composed of two hydrophones suspended in water at a certain depth by using the buoy and weights, and applied to acoustic signal acquisition of moving targets on or under water surface. Under the influence of Doppler generated by relative motion of the moving target and the passive sonar array, the received acoustic signals emitted from the target become non-stationary and time-varying. In this case, there must be significant errors between the true values of target detection or localization and the estimation results obtained by the methods based on stationary signal hypothesis.

Sonar (sound navigation and ranging) can be regarded as a kind of underwater radar, using sound to interrogate the surroundings. In general, sonar can be grouped into two main categories, i.e., active sonar and passive sonar, according to the presence and absence of a sound-projecting transducer as a component of the sonar system [1]. Passive sonar only includes a receiver without a transmitter and is used to detect the sound emitted by the target, which has the advantages of high concealment and a long detection distance. Therefore, passive sonar is widely used to detect, locate, and classify different targets on or under the surface of water, such as vessels and submarines [2,3], as illustrated in Figure 1. 

In the common scenario of passive target detection shown in Figure 1, the Doppler phenomenon, caused by relative motion between the sonar array and the moving target, can give rise to the distortion of received signals. Under the influence of Doppler, the received acoustic signals become non-stationary and time-varying. If the detection or localization of the moving target is operated by methods based on stationary signal hypothesis, the estimated results will definitely deviate from the true values. Therefore, it is necessary to utilize a signal model in which the influence of Doppler is taken into account. 

It is known that deterministic mechanical sounds and random hydrodynamic noise are the main components of underwater acoustic signals radiated by vessels [4]. Generally, a typical ship-radiated acoustic signal consists of a continuous spectrum and narrowband discrete components, which are known simply as lines or tonals, and can be successfully used for ship detection and classification. 

For the passive detection of ships, a spectrogram is often used to estimate the parabolic patterns and tonals in ship-radiated acoustic signals [5]. Moreover, performance of this kind of classical spectral estimation method based on Fourier transform, such as frequency resolution [6] and processing gain, is affected by the length of data used for spectral estimation. However, as for the non-stationary signal, its satisfactory spectral estimation result cannot be acquired from the long-time integration.

For non-stationary signal processing, time–frequency analysis [7,8] is widely used to provide the joint distribution information of the time and frequency domains, and describe the relationship between signal frequency and time. In addition, based on the assumption that the signal is stationary in a short time duration, high-resolution spectral estimation methods can be used to obtain the spectrum with high frequency resolution using a short time series. In this way, the high-resolution spectral estimation methods based on parametric models, such as autoregressive (AR), autoregressive moving average (ARMA) [9] and Multiple Signal Classification (MUSIC) [10] have been introduced to extract the narrowband characteristics of ship-radiated acoustic signal. In recent years, compressed sensing (CS) [11] has also been taken into account for high-resolution and precision extraction of the narrowband components of acoustic signals. However, the performances of these parametric spectral estimation techniques are limited by signal-to-noise ratio (SNR). 

An adaptive line enhancer (ALE) [12] is a classical method for enhancing the underwater narrowband discrete components (known as lines or tonals) in time-varying channels. However, the ability of ALE to deal with colored Gaussian noise is limited, and its performance deteriorates under lower SNR conditions [13]. In order to effectively extract or enhance the narrowband signals in complex environments, the conventional ALE is always used with a combination of other methods. For example, the incorporation of an $l_{1}$-norm sparse penalty into frequency domain adaption has been proposed in [14] to reduce the misadjustment of conventional ALE. Coherent integration and high-order cumulants have been combined with a multilevel switching adaptive line enhancer to obtain better performance [15]. 

Coherent integration [16] is essentially a digital filtering process to filter out much of the wideband noise, which is widely used in radar signal processing as an effective method for signal enhancement. However, the detection performance of coherent integration can be affected significantly by target motion, for instance, the imaging quality, target tracking, and identification [17]. It has become a highlight topic in radar signal processing to achieve long-time coherent integration for weak signals echoed by moving targets. Based on Fourier transform, the improved axis rotation discrete chirp-Fourier transform [18] and modified radon Fourier transform [19] were proposed to realize the compensations of range and Doppler migrations. In addition, a long-time coherent integration algorithm was proposed to compensate the phase of original echo signal by extracting the relative acceleration information carried in the echo signal, such that the compensated signals can be refocused in the frequency domain [20]. 

Similar to the application of coherent integration in radar signal processing, the SNR improvement of passive sonar signal processing that benefits from long-time coherent integration is also limited by the target motion. In order to increase the SNR, a coherent integration method of the cross-power spectrum with compensations of Doppler and time delay is proposed in this paper. The proposed method is implemented using the signals received by two hydrophones. In consideration of the Doppler and time delay between the two hydrophones, the received signal model is established and the cross-power spectrum of the received signals is derived. The Doppler factor and time delay can be estimated from the amplitude and phase of the cross-power spectrum. Then, the frequency shift and phase change in the cross-power spectrum can be corrected with the compensations of the estimated Doppler factor and time delay. Finally, the long-time coherent integration can be achieved by the compensated cross-power spectra of different time intervals. With this method, we can take full advantage of long-time data processing to get enough SNR gain for the weak and moving target detection. Compared with other methods, it is shown that the proposed method is capable of providing sufficient processing gain and effectively enhancing the narrowband discrete components in a ship-radiated acoustic signal. This is also of great significance for target recognition [21,22] and classification [23].

The paper is organized as follows. Section 2 introduces the signal model and the theory of coherent integration for the cross-power spectrum compensated with time scale factor and time delay. Different algorithms for discrete spectral estimation in simulations and experimental signal processing are analyzed in Section 3 and Section 4. Section 5 is exclusively dedicated to the conclusions.

## 2. Theory of Coherent Integration for the Cross-Power Spectrum with Doppler and Time Delay Compensations

### 2.1. Signal Model and Cross-Power Spectrum Coherent Integration

It has been mentioned in Introduction that the received signals from the passive sonar array are time-varying in the scenario illustrated in Figure 1. Therefore, it is necessary to establish the received signal model at first. The signals received by the two hydrophones are expressed as $r_{1}\left(n\right)$ and $r_{2}\left(n\right)$. Their subscripts represent the hydrophone numbers. When a moving target passes over these hydrophones, the target motion corresponding to each hydrophone results in a relative time scaling between the data received by these hydrophones. Thus, the time scale factor and time delay of the sound source signal received by one hydrophone can be modeled as a constant compared with the signal received by the other hydrophone over a short time interval. The received signals $r_{1}\left(n\right)$ and $r_{2}\left(n\right)$ can be written as

(1) $$\begin{array}{l} r_{1}\left(n\right)=s\left(n\right)+n_{1}\left(n\right)\\ r_{2}\left(n\right)=s\left(\frac{n-\tau }{\alpha }\right)+n_{2}\left(n\right) \end{array},$$

where s(n) is the sound source signal, τ is the time delay of the two received signals, α is $r_{2}\left(n\right)$’s time scale factor relative to $r_{1}\left(n\right)$. In the practical situation, $n_{1}\left(n\right)$ and $n_{2}\left(n\right)$ consist of the self-noise of the corresponding hydrophone and the complex environmental noise. Moreover, Moschas and Stirios [24] have confirmed that the outputs of hydrophones are different under the influence of dynamic noise of different types even if they are recording the same source excitation under the same propagation effects. However, the spectrum level of hydrophone self-noise can be designed to be ~20 dB smaller than the environmental noise for underwater detection [25]. Therefore, $n_{1}\left(n\right)$ and $n_{2}\left(n\right)$ are only the environmental noises received by each hydrophone and the self-noise of the hydrophone is not taken into account. 

In Equation (1), time scale factor α and time delay τ change with the movement of the sound source. If the two received signals are divided into M N-point length data segments, it is supposed that the speed of the moving target, the time scale factor α, and the time delay τ in each data segment remain constant. Then, the time scale factor and time delay can be rewritten as a=1−1/α, b=τ/α [26] in each data segment. Not only are the time scales of signals received by the two hydrophones different under the influence of Doppler, but the time scale factor of the signal received by each hydrophone also differs with sound source movement. The first data segment in $r_{1}\left(n\right)$ is set to $r_{11}\left(n\right)$, and its time scale factor is $\alpha_{11}=1$, i.e., $a_{11}=0$. The time scale factors of other data segments are represented according to $a_{11}$, and the i-th data segment of N-point length received by two hydrophones can be expressed as

(2) $$\begin{array}{l} r_{1i}\left(n\right)=s_{1i}\left(n-a_{1i}n\right)+n_{1i}\left(n\right)\\ r_{2i}\left(n\right)=s_{2i}\left[n-\left(a_{2i}n+b_{i}\right)\right]+n_{2i}\left(n\right)\\ n=0,1,\ldots ,N-1_{}\;i=1,2,\ldots ,M \end{array},$$

where $a_{1i}$ and $a_{2i}$ are the time scale factors of $r_{1i}\left(n\right)$ and $r_{2i}\left(n\right)$ relative to $r_{11}\left(n\right)$, respectively, and $b_{i}$ is the time delay between $r_{1i}\left(n\right)$ and $r_{2i}\left(n\right)$. In addition, $b_{i}=\tau_{i}/\alpha_{i}$, $\alpha_{i}$ is the time scale factor of $r_{2i}\left(n\right)$ relative to $r_{1i}\left(n\right)$. $n_{1i}\left(n\right)$ and $n_{2i}\left(n\right)$ are the noises in the i-th data segment received by the two hydrophones. The received signal model of each data segment for coherent integration is expressed in Equation (2).

For convenient deduction and analysis, the sound source is supposed to be a harmonic signal at $f_{0}$. The sampling interval is $△t_{s}$. Then, the received sound source signals in Equation (2) can be represented as

(3) $$\begin{array}{l} s_{1i}\left(n\right)=\exp\left[j2\pi f_{0}\left(n-a_{1i}n\right)△t_{s}\right]\\ s_{2i}\left(n\right)=\exp\left\{j2\pi f_{0}\left[n△t_{s}-\left(a_{2i}n△t_{s}+b_{i}\right)\right]\right\}\\ n=0,1,\ldots ,N-1_{}\;i=1,2,\ldots ,M \end{array}.$$

Then, the N-point length harmonic signal cross-power spectrum $P_{s12i}\left(f\right)$ of the i−th data segments $s_{1i}\left(n\right)$ and $s_{2i}\left(n\right)$ can be indicated as

(4) $$\begin{array}{ll} P_{s12i}\left(f\right) & =\left(\sum_{n=0}^{N-1}e^{j2\pi f_{0}\left(n-a_{1i}n\right)△t_{s}}e^{-j2\pi fn△t_{s}}\right){\left(\sum_{n=0}^{N-1}e^{j2\pi f_{0}\left[n△t_{s}-\left(a_{2i}n△t_{s}+b_{i}\right)\right]}e^{-j2\pi fn△t_{s}}\right)}^{*}\\ & =N^{2}\frac{\mathrm{sinc}\left(\pi N△t_{s}F_{1i}\right)}{\mathrm{sinc}\left(\pi △t_{s}F_{1i}\right)}\frac{\mathrm{sinc}\left(\pi N△t_{s}F_{2i}\right)}{\mathrm{sinc}\left(\pi △t_{s}F_{2i}\right)}e^{j\pi f_{0}\left[\left(N-1\right)△t_{s}\left(a_{2i}-a_{1i}\right)+2b_{i}\right]}\\ & =A_{i}\left(a_{1i},a_{2i}\right)e^{j\psi_{i}\left(a_{1i},a_{2i},b_{i}\right)} \end{array},$$

where $F_{1i}=\left(1-a_{1i}\right)f_{0}-f$, $F_{2i}=\left(1-a_{2i}\right)f_{0}-f$, sinc(x)=sin(x)/x, and ∗ is the symbol of conjugate operation. $A_{i}\left(a_{1i},a_{2i}\right)$ and $\psi_{i}\left(a_{1i},a_{2i},b_{i}\right)$ are the amplitude and phase of $P_{s12i}\left(f\right)$, and can be represented as

(5) $$\begin{array}{l} A_{i}\left(a_{1i},a_{2i}\right)=N^{2}\frac{\mathrm{sinc}\left(\pi N△t_{s}F_{1i}\right)}{\mathrm{sinc}\left(\pi △t_{s}F_{1i}\right)}\frac{\mathrm{sinc}\left(\pi N△t_{s}F_{2i}\right)}{\mathrm{sinc}\left(\pi △t_{s}F_{2i}\right)}\\ \psi_{i}\left(a_{1i},a_{2i},b_{i}\right)=\pi f_{0}\left[\left(N-1\right)△t_{s}\left(a_{2i}-a_{1i}\right)+2b_{i}\right] \end{array}.$$

It is evident from Equation (5) that the amplitude $A_{i}\left(a_{1i},a_{2i}\right)$ of $P_{s12i}\left(f\right)$ is actually affected by the frequency shift generated by Doppler (time scale factor), and the phase $\psi_{i}\left(a_{1i},a_{2i},b_{i}\right)$ is strictly under the influence of time delay and time scale factor. Although the changes in amplitude and phase with sound source movement make impossible the coherent integration of $P_{s12i}\left(f\right)$; the cross-power spectrum coherent integration can be realized by compensations of time scale factor and time delay. 

### 2.2. Estimations and Compensations of Doppler Factor and Time Delay for Cross-Power Spectrum

#### 2.2.1. Methods for Doppler Factor and Time Delay Estimations

For the estimations of time scale (Doppler) factor and time delay, the joint estimation method of time scale factor and time delay based on the cross-ambiguity function [27,28] is widely employed in both radar and sonar domains. The cross-ambiguity function of the received signals $r_{1}\left(n\right)$ and $r_{2}\left(n\right)$ can be expressed as

(6) $$W_{r_{2}r_{1}}\left(a,b\right)=\sqrt{1-a}\sum_{n=1}^{N}r_{1}\left(n\right)r_{2}{}^{T}\left(n-an-b\right){,}_{}{}_{}a<1$$

where $W_{r_{2}r_{1}}\left(a,b\right)$ is the cross-ambiguity function, a and b are time scale factor and time delay, respectively. The symbol T represents transpose. With the defined search ranges of a and b, we can obtain a two-dimensional plane of the cross-ambiguity function $W_{r_{2}r_{1}}\left(a,b\right)$. The time scale factor and time delay corresponding to the maximum of $W_{r_{2}r_{1}}\left(a,b\right)$ are the estimation results, i.e.,

(7) $$\left(\bar{a},\bar{b}\right)=\arg\left\{\underset{a,b}{\max}W_{r_{2}r_{1}}\left(a,b\right)\right\},$$

where $\bar{a}$ and $\bar{b}$ are the estimation results of time scale factor and time delay.

However, the joint estimation method requires a considerable amount of calculations for maximum outcomes on a two-dimensional plane. In addition, it is difficult to obtain the maximum on this two-dimensional plane when the target is moving at a low speed, even if the search step size is enough. In order to estimate the time scale factor and time delay quickly and accurately, a two-step method proposed in our previous work [29] can be used. Compared with the joint estimation method, the time scale factor and time delay are estimated step by step. First, a one-dimensional search is performed for the estimation of time scale factor. Then, the time delay can be obtained by the phase of the cross-power spectrum compensated with the estimated time scale factor. This two-step method reduces the search dimension, which can avoid a large amount of calculations.

The detailed principle and implementation of this two-step method can be seen in Appendix A. 

#### 2.2.2. Compensations of Doppler Factor and Time Delay for Cross-Power Spectrum

**Figure 2:**
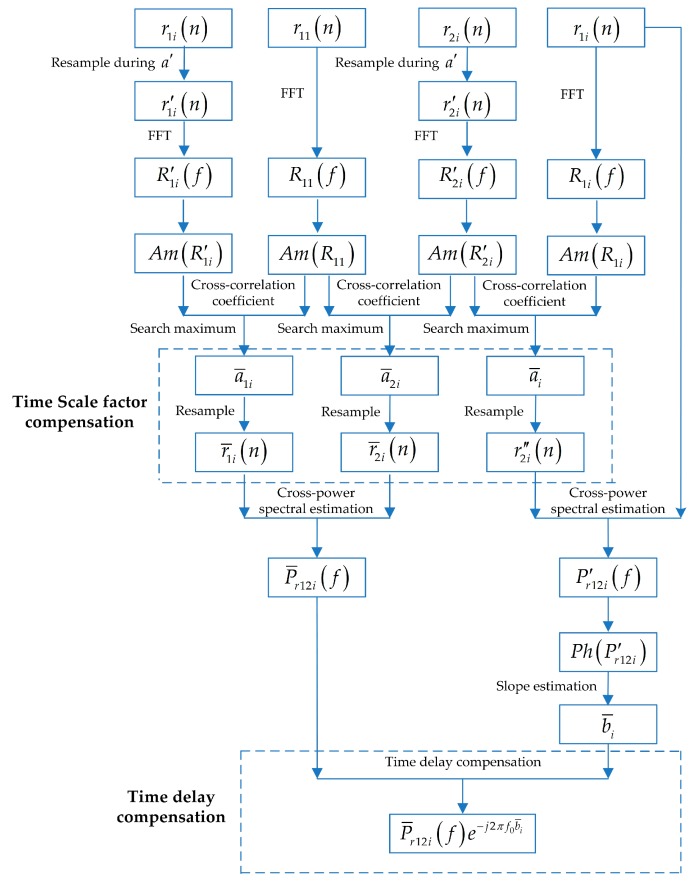
The flow chart for time scale factor and time delay compensations of cross-power spectrum.

Several methods for the estimations of time scale factor and time delay have been mentioned in the previous section. The specific compensation process of time scale factor and time delay, aimed at the cross-power spectrum, is illustrated in Figure 2. The two-step method demonstrated in Appendix A is used to estimate the time scale factor and time delay in Figure 2. In fact, the time scale factor and time delay estimated by any method can be used for the compensations of the cross-power spectrum according to the operations illustrated in the dashed rectangles of Figure 2.

As illustrated in Figure 2, $a^{\prime }$ is the search range of time scale factor. $r_{1i}^{\prime }\left(n\right)$ and $r_{2i}^{\prime }\left(n\right)$ are the resampling results of $r_{1i}\left(n\right)$ and $r_{2i}\left(n\right)$, respectively, in the range of $a^{\prime }$. The amplitudes $Am\left({R^{\prime }}_{1i}\right)$ and $Am\left({R^{\prime }}_{2i}\right)$ are obtained from $R_{1i}^{\prime }\left(f\right)$ and $R_{2i}^{\prime }\left(f\right)$, i.e., the FFT (fast Fourier transform) results of $r_{1i}^{\prime }\left(n\right)$ and $r_{2i}^{\prime }\left(n\right)$. The amplitude $Am\left(R_{11}\right)$ of $r_{11}\left(n\right)$ can be acquired in the same way. Then, we proceed with the calculations of the cross-correlation coefficients of $Am\left(R_{1i}^{\prime }\right)$ and $Am\left(R_{11}\right)$, as well as $Am\left(R_{2i}^{\prime }\right)$ and $Am\left(R_{11}\right)$. The time scale factors ${\bar{a}}_{1i}$ and ${\bar{a}}_{2i}$ of $r_{1i}\left(n\right)$ and $r_{2i}\left(n\right)$, relative to $r_{11}\left(n\right)$, can be estimated by searching the time scale factors in $a^{\prime }$ corresponding to the maxima of cross-correlation coefficients. $r_{1i}\left(n\right)$ and $r_{2i}\left(n\right)$ are resampled again according to ${\bar{a}}_{1i}$ and ${\bar{a}}_{2i}$ to acquire ${\bar{r}}_{1i}\left(n\right)$ and ${\bar{r}}_{2i}\left(n\right)$. Then, the time scale factors of $r_{11}\left(n\right)$, ${\bar{r}}_{1i}\left(n\right)$, and ${\bar{r}}_{2i}\left(n\right)$ become the same. After the compensation of time scale factor, the frequencies of sound source estimated by each cross-power spectrum ${\bar{P}}_{r12i}\left(f\right)$ of ${\bar{r}}_{1i}\left(n\right)$ and ${\bar{r}}_{2i}\left(n\right)$ become the same, i.e., the frequency shifts are compensated with the estimated time scale factors.

Due to the difference between the time scale factors of $r_{1i}\left(n\right)$ and $r_{2i}\left(n\right)$, the time scale factors should be compensated prior to time delay estimation. As indicated in Figure 2, the time scale factor ${\bar{a}}_{i}$ of $r_{2i}\left(n\right)$ relative to $r_{1i}\left(n\right)$ can be estimated by assessing the value in $a^{\prime }$ corresponding to the maximum of cross-correlation coefficients between $Am\left(R_{1i}\right)$ and $Am\left(R_{2i}^{\prime }\right)$. Then, $r_{2i}^{\prime \prime }\left(n\right)$ can be obtained from the resampled $r_{2i}\left(n\right)$ according to ${\bar{a}}_{i}$, which has the same time scale factor as $r_{1i}\left(n\right)$. The phase $Ph\left(P_{r12i}^{\prime }\right)$ can be acquired from the cross-power spectrum $P_{r12i}^{\prime }\left(f\right)$ of $r_{1i}\left(n\right)$ and $r_{2i}^{\prime \prime }\left(n\right)$. The slope estimation of $Ph\left(P_{r12i}^{\prime }\right)$ is divided by 2π to obtain the time delay estimation result. Finally, the time delay of the sound source at $f_{0}$ in the cross-power spectrum ${\bar{P}}_{r12i}\left(f\right)$ can be compensated by multiplying by $e^{-j2\pi f_{0}{\bar{b}}_{i}}$.

### 2.3. Implementation of Coherent Integration Using the Compensated Cross-Power Spectrum and Analysis of Integration Gain 

After the aforementioned compensations of time scale factor and time delay highlighted in Figure 2, the frequency and phase of the sound source in each compensated cross-power spectrum ${\bar{P}}_{r12i}\left(f\right)e^{-j2\pi f_{0}{\bar{\tau }}_{i}}$ are entirely similar. The amplitude and phase of the compensated cross-power spectrum of sound source signal can be written as

(8) $${\bar{A}}_{i}=N^{2}\frac{{\mathrm{sinc}}^{2}\left[\pi N△t_{s}\left(f_{0}-f\right)\right]}{{\mathrm{sinc}}^{2}\left[\pi △t_{s}\left(f_{0}-f\right)\right]}{,}_{}{\bar{\psi }}_{i}=0.$$

According to Equation (8), the compensated cross-power spectrum of the sound source signal in each data segment can be represented as

(9) $${\bar{P}}_{s12i}\left(f\right)=\frac{N^{2}\sin c^{2}\left[\pi N△t_{s}\left(f_{0}-f\right)\right]}{\sin c^{2}\left[\pi △t_{s}\left(f_{0}-f\right)\right]}.$$

As is evident in Equation (9), the frequency estimation result of each compensated cross-power spectrum is analogous, and the phase estimation is transformed to zero. These outcomes satisfy the conditions of coherent integration. The coherent integration result of ${\bar{P}}_{s12i}\left(f\right)$ can be written as

(10) $${\bar{P}}_{s12}\left(f\right)=\sum_{i=1}^{M}\frac{N^{2}\sin c^{2}\left[\pi N△t_{s}\left(f_{0}-f\right)\right]}{\sin c^{2}\left[\pi △t_{s}\left(f_{0}-f\right)\right]}.$$

The incoherent integration obtained from the uncompensated cross-power spectra of the sound source signal illustrated in Equation (4) can be expressed as

(11) $$P_{s12}\left(f\right)=\sum_{i=1}^{M}\left|P_{s12i}\left(f\right)\right|=\sum_{i=1}^{M}N^{2}\left|\frac{\sin c\left(\pi N△t_{s}F_{1i}\right)}{\sin c\left(\pi △t_{s}F_{1i}\right)}\frac{\sin c\left(\pi N△t_{s}F_{2i}\right)}{\sin c\left(\pi △t_{s}F_{2i}\right)}\right|.$$

Compared with the incoherent integration result in Equation (11), the additional integration gain offered by the coherent integration shown in Equation (10) can be written as

(12) $$G=10\log_{10}\left(\frac{{\bar{P}}_{s12}\left(f\right)}{P_{s12}\left(f\right)}\right).$$

For the harmonic signal of $f=f_{0}$, the extra integration gain at $f_{0}$ is

(13) $$G_{f=f_{0}}=10\log_{10}M-10\log_{10}\left(\sum_{i=1}^{M}\left|\frac{\sin c\left(\pi N△t_{s}a_{1i}f_{0}\right)}{\sin c\left(\pi △t_{s}a_{1i}f_{0}\right)}\frac{\sin c\left(\pi N△t_{s}a_{2i}f_{0}\right)}{\sin c\left(\pi △t_{s}a_{2i}f_{0}\right)}\right|\right).$$

The aforementioned derivations suggest that the integration gain provided by the compensated cross-power spectra under ideal conditions is $10\log_{10}M$, which is proportional to the number of integrations. The performance of incoherent integration is limited by Doppler and phase differences in each data segment, which are responsible for the confined gain of incoherent integration. In the ensuing sections, the performance of the proposed method and the comparisons with other spectral estimation algorithms will be elaborated by employing certain simulations and experimental data processing.

## 3. Performance of Different Algorithms for Discrete Spectral Estimation in Simulation

### 3.1. Simulated Signals

**Figure 3:**
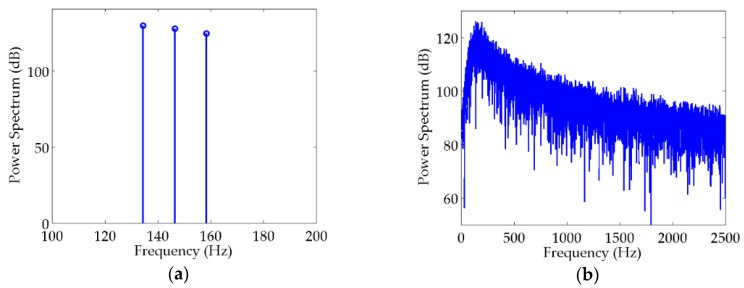
Simulation of ship-radiated acoustic signal: (**a**) discrete spectra; (**b**) continuous spectrum.

The method proposed in [30] was applied to simulate a ship-radiated acoustic signal in this section. The characteristics of the signal acquired through this particular method were fairly analogous to the practical ship properties. The frequencies of discrete spectra contained in the ship-radiated acoustic signal are set at 134, 146, and 158 Hz, corresponding to the power spectra of 130, 128, and 125 dB, respectively, which are illustrated in Figure 3a. The continuous spectrum was obtained from the output of white Gaussian noise processed through a bandpass filter with a sampling frequency of 10 kHz. The amplitude of the continuous spectrum increased with 2.82 dB/oct during 0–150 Hz, and decreased with −2.10 dB/oct at other frequencies. The maximum of the continuous spectrum was set as 125 dB at a frequency of 150 Hz, which can be seen in Figure 3b. The SNRs of discrete spectra in the simulated ship-radiated acoustic signal are 12, 5, and 3 dB.

**Figure 4:**
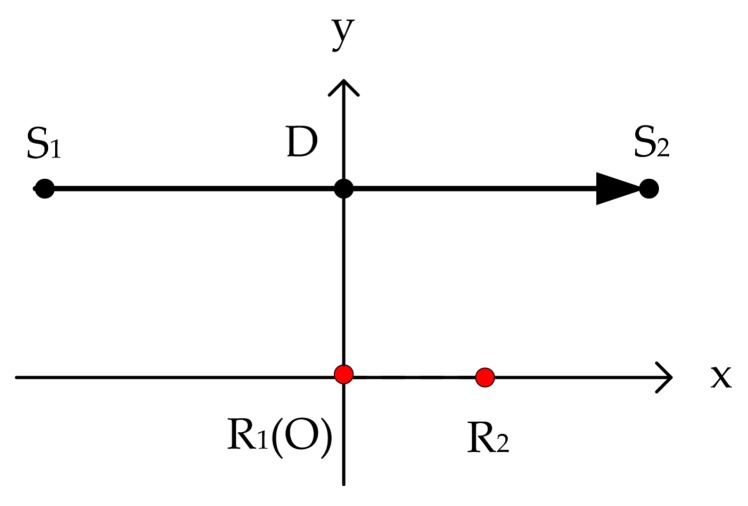
Side view of the geometric conditions for hydrophone deployment and ship track. The target ship sailed through the distance from S_1_ to S_2_ on the water surface. The hydrophones R_1_ and R_2_ were suspended at the depth of DO (vertical distance between the hydrophones and water surface) and floating parallel to the water surface for acoustic signal acquisition.

The received signals of two hydrophones were simulated using the ship-radiated acoustic signal mentioned above. The conditions for array deployment and ship navigation are indicated in Figure 4. The two hydrophones, placed 5 m apart, were assumed to be suspended at a depth of 150 m and floating parallel to the water surface. The target ship sailed through the distance from S_1_ to S_2_ at a speed of 10 m/s. The direct sound signals received by R_1_ and R_2_ were the simulated signals for performance analysis of different spectral estimation methods. Distortions of the received signals due to the hydrophones were not considered in the simulation.

### 3.2. Analysis of Narrowband Discrete Spectral Estimation Results Obtained from Different Methods

In this section, the algorithms listed in Table 1 were applied to the spectral estimation of the signals simulated in Section 3.1. 

**Figure 5:**
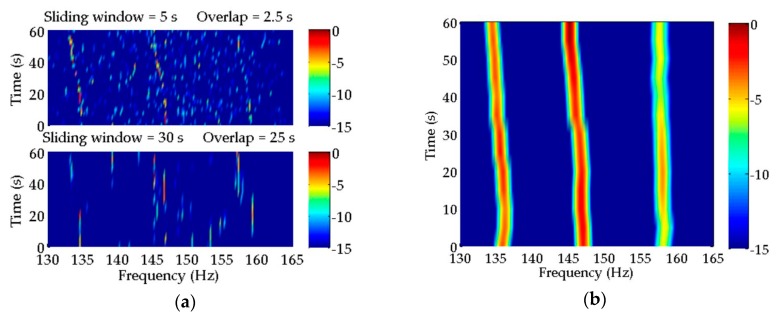

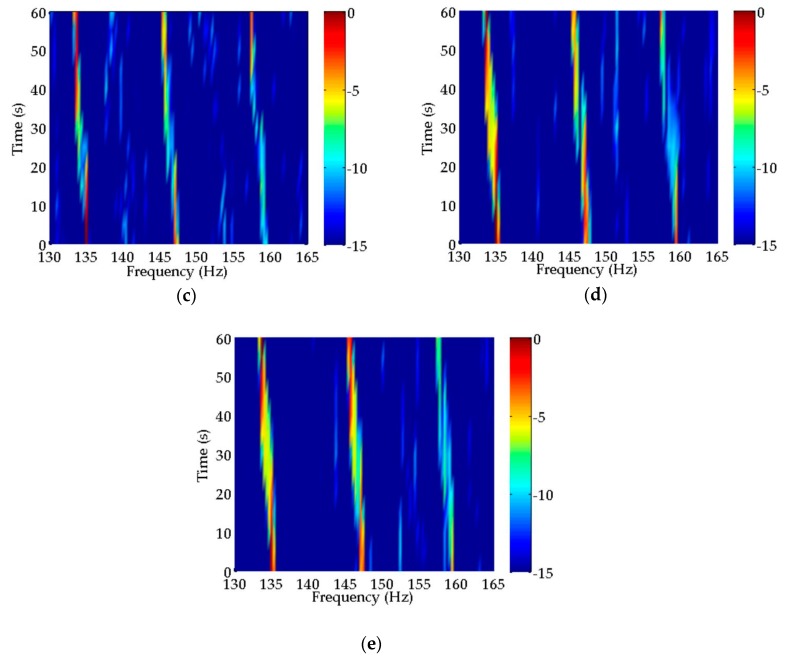
Estimation results of discrete spectra in simulated signals with SNR = 20 dB obtained by (**a**) compressed sensing (CS); (**b**) Multiple Signal Classification (MUSIC); (**c**) Cross-Power Spectrum Coherent Integration (CPSCI); (**d**) Cross-Power Spectrum Incoherent Integration (CPSII) and (**e**) Compensated Cross-Power Spectrum Coherent Integration (CCPSCI). (**a**) In the top subfigure, CS can clearly reflect the frequency changes in discrete spectra with time. However, the estimated discrete spectra are off and on, which is more serious in the estimation result of the bottom subfigure. (**b**) MUSIC can provide quite good estimation results of the two discrete spectra with high SNRs. However, the frequency change in the weakest discrete spectrum is estimated by mistake. In (**c**–**e**), CPSCI, CPSII and CCPSCI can estimate the frequencies of discrete spectra steadily and can represent the frequency variations accurately in comparison with CS and MUSIC. During the time period of ~20–50 s, the discrete spectra estimated by CPSCI and CPSII start to disconnect. However, the proposed CCPSCI can still offer relatively stable and continual spectral estimations.

**Figure 6:**
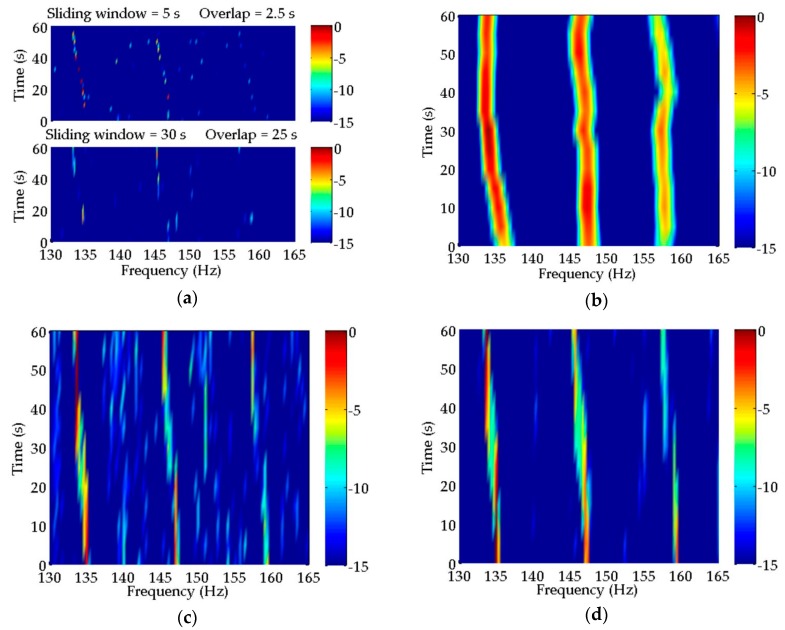

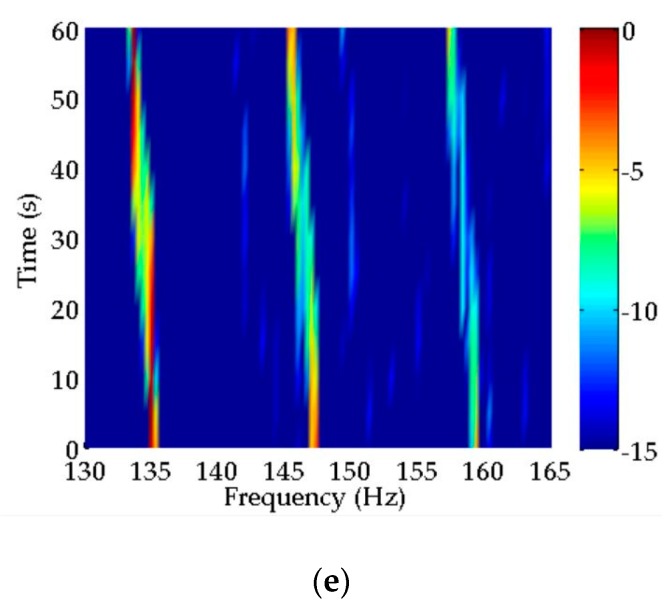
Estimation results of discrete spectra in simulated signals with signal-to-noise ratio (SNR) = −10 Db obtained by (**a**) CS; (**b**) MUSIC; (**c**) CPSCI; (**d**) CPSII and (**e**) CCPSCI. (**a**) In the estimation results of CS, the number of breaking points increases in both subfigures. The weakest discrete spectrum is hard to detect. (**b**) The decrease in SNR leads to the further increase in the frequency errors of discrete spectra estimated by MUSIC. In (**c**,**d**), the discontinuity of discrete spectra estimated by CPSCI and CPSII is obvious, which brings difficulties to target identification and classification. (**e**) CCPSCI can still provide the spectral estimation result with a relatively high degree of continuity and stability.

It is noted that Cross-Power Spectrum Coherent Integration (CPSCI) is the coherent integration of the cross-power spectrum without compensation. The methods listed in Table 1 are appropriate for spectral estimation under the condition that the background noise is assumed to be white Gaussian noise. The SNRs of ship-radiated acoustic signal to background noise were set at 20 dB and −10 dB, respectively. The estimation results obtained from the methods mentioned above are all displayed by low-frequency analysis and recording (LOFAR) and shown in Figure 5 and Figure 6. For the LOFAR processing of the methods listed in Table 1, the length of the sliding window is 30 s and the overlap is 25 s. In addition, the CS method is also used for LOFAR processing with a sliding window of 5 s and an overlap of 2.5 s, which is illustrated in Figure 5a and Figure 6a, corresponding to different SNRs. The specific process of these methods for spectral estimation can be seen in Appendix B.

As shown in Figure 5a, CS method can provide pretty high frequency resolution. In the top subfigure of Figure 5a, the CS method can perform well with a short sliding window to reflect the frequency changes in discrete spectra with time caused by the target motion. However, the estimation inaccuracy of some frequencies leads to the poor continuity of discrete spectra illustrated by LOFAR. Moreover, the spectral estimation result of CS is not satisfactory using a long sliding window. In the bottom subfigure of Figure 5a, it is difficult for us to identify the discrete spectra of moving target from the LOFAR result. This suggests that the frequency changes in a large amount of time-varying signal samples should be taken into consideration for a better spectral estimation result. Compared with CS, the estimation result of MUSIC in Figure 5b shows that MUSIC has a better tolerance for time-varying signal processing. Except for the weakest discrete spectrum, the time–frequency characteristics of the other two discrete spectra with higher SNRs can be reflected correctly in the LOFAR result of MUSIC.

The frequency changes indicated in the estimation results of CPSCI, CPSII, and CCPSCI in Figure 5c–e are not as clear as CS, due to the limited frequency resolution provided by them. However, these three methods can estimate the frequencies of discrete spectra with a fair amount of stability and can represent the circumstance of frequency variation accurately in comparison with CS and MUSIC. During the time period in which the frequencies of discrete spectra change rapidly with time (~20–50 s), the discrete spectra estimated by CPSCI and CPSII start to disconnect because the integration gains provided by them are insufficient for extracting the weak discrete spectra at this time. In contrast to CPSCI and CPSII, CCPSCI (with the compensations of Doppler factor and time delay) may offer to estimate weak discrete spectra, even for abrupt variations in frequency. In this way, our method can offer relatively stable and continual discrete spectral estimation results.

The estimation results of these methods with a low SNR of −10 dB are shown in Figure 6. The number of breaking points in the discrete spectra, estimated by CS in Figure 6a, increases so much that the weakest discrete signal is almost undetectable in both subfigures. In addition, the decrease in SNR causes the frequency errors of discrete spectra estimated by MUSIC in Figure 6b to increase even further. As for CPSCI and CPSII in Figure 6c–d, there are obvious gaps in the discrete spectra, which can make target detection and identification almost impossible in real scenarios. Moreover, the performance of CPSCI using traditional coherent integration significantly deteriorates due to the influences from frequency and phase changes under a low SNR. The comparative outcomes of Figure 6e illustrate that CCPSCI can still provide the spectral estimation result with a relatively high degree of continuity and stability, even at a low SNR.

Based on the comprehensive analysis of the above methods, we may summarize the conclusions in the following section. Firstly, the performance of the classical CS algorithm for discrete spectral estimation depends, to some extent, on SNR and the premise of a stationary signal, and it may not be able to detect the weak discrete spectrum in real scenarios. Similarly, the estimation error of the discrete spectrum obtained from MUSIC increases significantly when the SNR decreases. Although increasing the number of snapshots can improve the processing performance of the MUSIC algorithm [31], the calculation time would also increase dramatically at the same time [32]. Compared to CS and MUSIC, more stable spectral estimation results can be provided by CPSCI, CPSII, and CCPSCI, which are based on Fourier theory. However, under the influence of Doppler phenomenon, the integration gains obtained from CPSCI and CPSII are so limited that the discrete spectra cannot be detected during this period. Subsequently, the resulting discontinuous discrete spectrum has a negative impact on target recognition. By contrast, the proposed CCPSCI can effectively overcome the influence of Doppler on integration gain with Doppler factor and time delay compensations. This, in turn, allows the coherent integration of the cross-power spectrum. Consequently, the result of the discrete spectrum estimated by CCPSCI has the advantages of being stable, continual, and accurate throughout the detection process. 

In addition to the evaluation of spectral estimation performance discussed above, the calculation time required during spectral estimation is also an important reference for measuring the engineering application value of the method. It takes hours for us to obtain the spectral estimation results by CS and MUSIC in the simulations. However, the methods based on classical spectral estimation theory, CPSCI, CPSII, and CCPSCI require much less calculation time than either CS or MUSIC. Although the proposed CCPSCI requires a bit of extra time (several minutes) for spectral estimation as compared to CPSCI and CPSII (less than 1 second), the calculation of CCPSCI can be further reduced with an improved estimation process for the time scale factor in our future research. 

### 3.3. Extra Integration Gain

**Figure 7:**
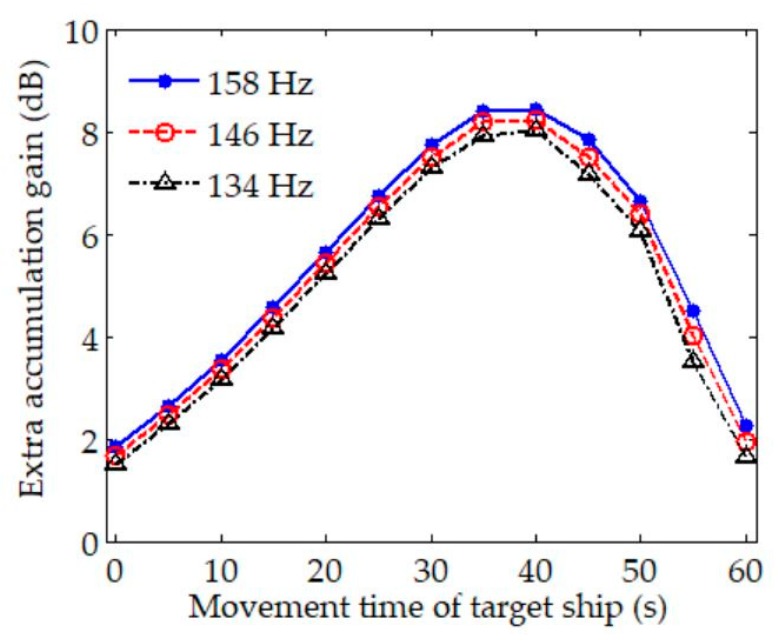
Extra integration gain provided by CCPSCI compared with CPSII in simulation.

As mentioned above, coherent integration can provide more integration gain than incoherent integration. Under the simulation conditions of Section 3.1 and Section 3.2, the extra integration gain provided by CCPSCI compared with CPSII can be calculated according to Equation (13) in Section 2.3, and is illustrated in Figure 7. 

The extra integration gains provided by CCPSCI for discrete spectra with frequencies of 134, 146, and 158 Hz are shown in Figure 7. The horizontal axis of this figure represents the movement time of the target ship, which is equivalent to the vertical axis of LOFAR in Figure 5 and Figure 6, i.e., the time axis. In Figure 7, extra integration gain varies with the movement time of the target ship because the time scale factor changes with the target motion. It can be known from the simulation environment and spectral estimation results that frequency shifts are very obvious during the time period of ~20–50 s under the influence of Doppler. During this period, CCPSCI can offer a ~6–8 dB extra integration gain compared with CPSII. As shown in Figure 7, the extra integration gains of these three discrete spectra are slightly different. Under the same conditions, the frequency shift in the higher frequency discrete spectrum is bigger, so the performance of incoherent integration is worse. Therefore, the proposed CCPSCI method can provide extra integration gains for the discrete spectrum at a higher frequency.

**Figure 8:**
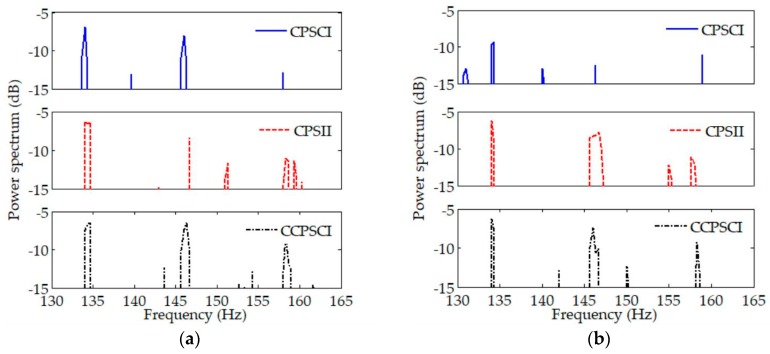
Averages of power spectra estimated by CPSCI, CPSII, and CCPSCI with (**a**) SNR = 20 dB and (**b**) SNR = −10 dB during the time period of ~20–50 s in simulation. Compared with CPSII and CCPSCI, the performance of CPSCI for signal enhancement is unsatisfactory and gets worse under low SNR. As shown in (**a**,**b**), CCPSCI can provide an extra integration gain of ~2–4 dB.

The averages of power spectra estimated by CPSCI, CPSII, and CCPSCI during the time period of ~20–50 s were calculated using the normalized spectral estimation results in the corresponding time period in Figure 5 and Figure 6, which can be seen in Figure 8. We ignored the frequency changes in discrete spectra when calculating the averages. The value of the power spectrum corresponding to each discrete spectrum is listed in Table 2. 

Through the comparison and analysis of the averages of power spectra shown in Figure 8 and Table 2, it can be seen that the performance of CPSCI for signal enhancement is unsatisfactory. The processing gain provided by CPSCI is particularly limited due to the influence from Doppler under low SNR. Compared with CPSII and CCPSCI, the frequency and phase errors in CPSCI cause a loss of integration gain. However, the proposed CCPSCI can reduce this loss by correcting the frequency shifts and phase changes in the cross-power spectrum. As shown in Table 2, CCPSCI can provide an additional ~2–4 dB integration gain compared with other methods.

## 4. Analysis of Experimental Data Processing Using Different Methods

The simulations in Section 3 analyze the discrete spectral estimation performance of CS, MUSIC, CPSCI, CPSII, and CCPSCI. In this section, these methods are applied to experimental data processing and evaluations are based on the estimation outcomes. The experimental data used for the analysis were collected in an oceanic experiment. The passive sonar array was deployed at a depth of 170 m and the element spacing was kept at 0.3 m. The data received by two adjacent hydrophones of that particular sonar array were used for signal processing. In order to compare the performance of these methods conveniently, the data of the target ship cruising in the proximity of the array at a speed of 12 knots (approximately 6 m/s) were selected for discrete spectral estimation. 

**Figure 9:**
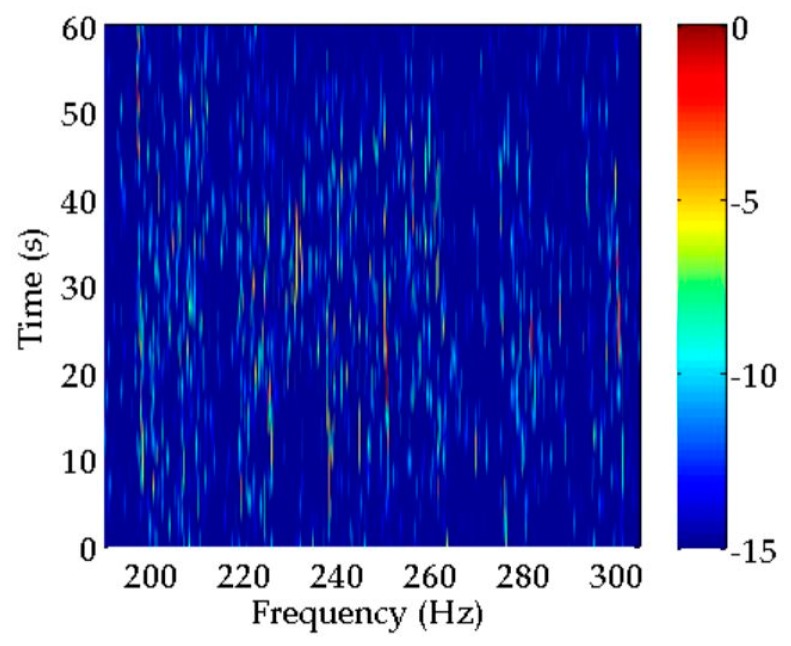
Spectral estimation result obtained by a periodogram.

A classical spectral estimation method, i.e., periodogram, is used for LOFAR to process the data from one hydrophone, in which the sliding window is 5 s and the overlap is 2.5 s. The LOFAR result estimated by the periodogram is displayed in Figure 9. This LOFAR result can help us to achieve comparative analysis among the various spectral estimation methods discussed in the previous section.

**Figure 10:**
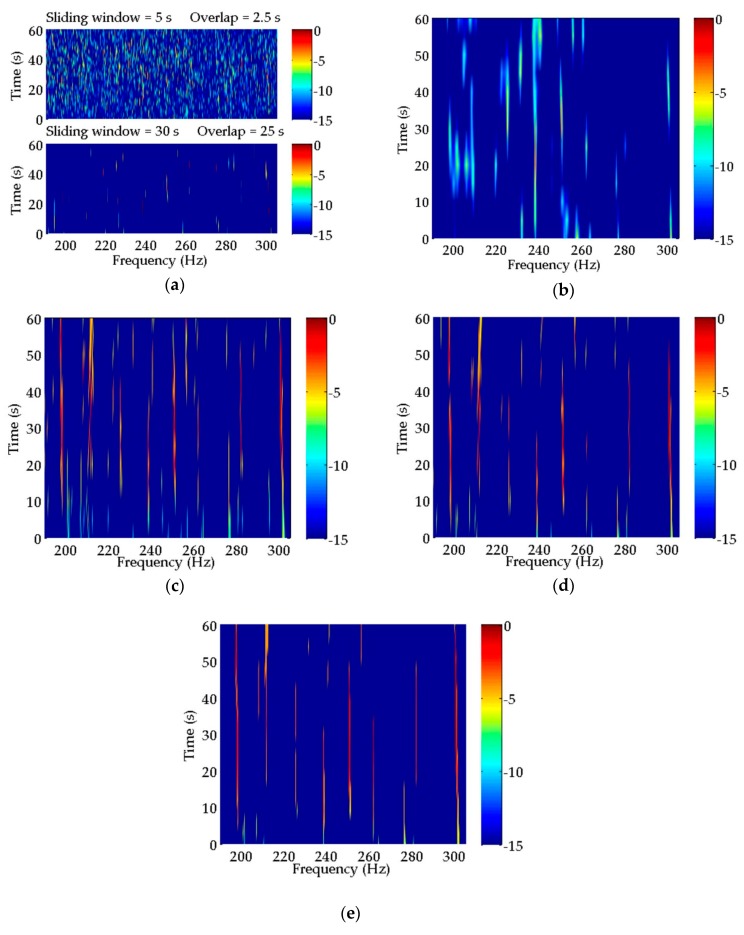
Spectral estimation results of the experimental data obtained by (**a**) CS; (**b**) MUSIC; (**c**) CPSCI; (**d**) CPSII; and (**e**) CCPSCI. Compared with the periodogram, a few discrete spectra can be enhanced by CS. However, it is difficult to extract the characteristic discrete spectra from the CS spectral estimation results in both subfigures. In the estimation result from MUSIC, the discrete spectra at the frequencies of 225, 238, and 251 Hz have an octave relationship. In (**c**–**e**), the LOFAR results of CPSCI, CPSII and CCPSCI are much improved in comparison to CS and MUSIC. The discrete spectra at frequencies of 199, 212, 225, 238, 251 and 301 Hz obtained from these three methods can be regarded as the characteristic discrete spectra of the target.

As indicated in Figure 9, there are many discrete spectra that can be extracted by a periodogram in the current time period and frequency band. However, it is very difficult to determine which discrete spectra belong to the target ship in this particular situation, where multifarious discrete spectra mingle without any regular features. Therefore, it is necessary to process the received data further. The algorithms mentioned in Table 1 are applied to data processing, and their LOFAR results are illustrated in Figure 10. For all the methods, LOFAR processing is operated with a sliding window of 30 s and an overlap of 25 s. In addition, the LOFAR results obtained by CS using two different sliding windows are shown in Figure 10a. The detailed process of these methods for spectral estimation of experimental data can be seen in Appendix B.

Compared with the periodogram, the background noise in the estimation results of CS illustrated in Figure 10a using the sliding window of 5 s is more obvious. In the bottom subfigure of Figure 10a, the background noise is much reduced using a longer sliding window. Although a few discrete spectra have been enhanced in comparison with the periodogram, it is still hard to detect and identify characteristic discrete spectra from the CS spectral estimation results. 

As for the result estimated by MUSIC in Figure 10b, in the frequency band of ~220–260 Hz, there are discrete spectra at the frequencies of 225, 238, and 251 Hz. In addition, these discrete spectra have an octave relationship. However, they have short durations and are simultaneously affected by other spectra. Thus, we may only deduce that these discrete spectra are probably the characteristic tonal spectra of the target ship.

The LOFAR results of CPSCI, CPSII, and CCPSCI in Figure 10c–e are significantly improved in comparison to CS and MUSIC, which is reflected in the reduction in background noise and the enhancement of the discrete spectrum. Similar to the simulation results, the integration gains obtained from CPSCI and CPSII are decreased during the time period in which the frequencies of the discrete spectra change rapidly, which leads to the discontinuity of discrete spectrum illustrated by LOFAR. As a result, it is not suitable for CPSCI and CPSII to estimate the weak discrete spectrum severely affected by Doppler. In order to extract the weak discrete spectrum under the influence of Doppler, the CCPSCI algorithm based on the compensations of time scale factor and time delay can play an important role. This is attributed to our proposed method providing sufficient integration gain by correcting the frequency shifts and phase changes in the cross-power spectrum. Therefore, the discrete spectrum obtained from CCPSCI has a good quality of continuity, which helps to track the target ship and aids in comprehensible identification. As shown in Figure 10e, the discrete spectra at frequencies of 199, 251, and 301 Hz have the strongest energy, longest duration, and obvious frequency shifts with time. These discrete spectra may be regarded as the characteristic discrete spectra influenced by the diesel engine, shaft, and valve piston of the target ship, respectively. Simultaneously, there are five discrete spectra in the frequency band of ~190–270 Hz showing a relationship of 13 Hz octave, which represents the ignition frequency characteristic of a diesel engine.

**Figure 11:**
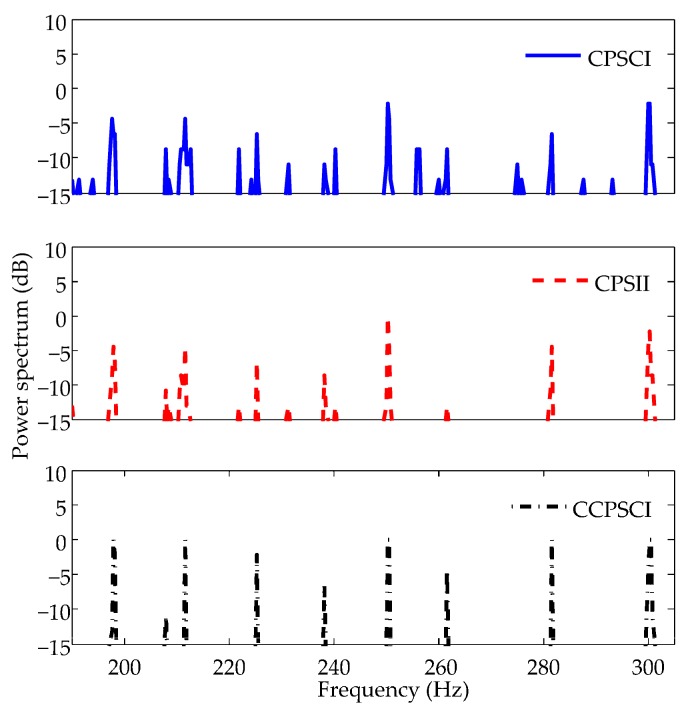
Averages of power spectra estimated by CPSCI, CPSII, and CCPSCI in experimental data processing. It can be seen from these results that CPSII and CCPSCI can improve the SNRs of discrete spectra much better than CPSCI. However, the discrete spectrum at 262 Hz cannot be detected by CPSII. Compared with CPSCI and CPSII, CCPSCI can provide an additional 2~6 dB integration gain.

In Figure 10c–e, the averages of power spectra estimated by CPSCI, CPSII, and CCPSCI are calculated during the time period of ~20–40 s. The averages of power spectra are shown in Figure 11, and the exact averages of discrete spectra are listed in Table 3.

It can be seen in Figure 11 that the interference of undesired discrete spectra in the spectral estimation result of CPSCI is stronger than CPSII and CCPSCI. It is easier for us to identify the characteristic discrete spectra in the spectral estimation results of CPSII and CCPSCI. Seen from the averages of the discrete spectra in Table 3, it can be concluded that CCPSCI can provide an additional 2~6 dB integration gain compared with CPSCI and CPSII. Consequently, this makes it appropriate for the proposed method to be applied to target detection in time-varying coherent channels.

## 5. Conclusions

In order to estimate the narrowband discrete components of ship-radiated acoustic signals for target detection, a coherent integration method of a cross-power spectrum with time scale factor and time delay compensations was proposed in this paper. The data received by two hydrophones were modeled and applied to cross-power spectrum estimation. With the estimation results of time scale factor and time delay, the frequency shifts and phase changes existing in the cross-power spectra can be compensated. Thus, we were capable of performing coherent integration with these compensated cross-power spectra in order to enhance and extract the discrete spectra in the ship-radiated acoustic signal. The performance of CS, MUSIC, cross-power spectrum coherent integration (CPSCI), cross-power spectrum incoherent integration (CPSCII), and the proposed method compensated cross-power spectrum coherent integration (CCPSCI) were analyzed in simulations and experimental data processing. The conclusions are summarized as follows.

The classical CS method used in this paper is not appropriate for spectral estimation at a low SNR or with the long time duration of a non-stationary signal. The performance of MUSIC can be enhanced by utilizing more snapshots. However, longer data contain more variations in frequency, which may restrict the improvement obtained through increasing the number of snapshots.

When the frequencies of discrete spectra fluctuate greatly under the influence of Doppler, the integration gain provided by CPSCI and CPSII is too low to estimate the discrete spectrum. The phase error in CPSCI can also cause a loss of integration gain. The frequency shifts and phase changes in the cross-power spectrum can be corrected by the proposed method (CCPSCI) to provide higher integration gain satisfactorily under the influence of Doppler.

Our proposed method has advantages in spectral estimation, calculation time, and Doppler compensation. It can be applied to longer duration coherent integration to enhance and estimate the weak narrowband signals in ship-radiated acoustic signals in a time-varying coherent channel. Moreover, the proposed method can provide the feasible and convincing estimation results of a discrete spectrum and can be easily demonstrated in engineering applications, which assures certainty for passive detection and timely warning during real-time scenarios.

## Figures and Tables

**Table 1:** Methods for spectral estimation and their abbreviations.

Algorithm	Abbreviations
Compressed Sensing	CS
Multiple Signal Classification	MUSIC
Cross-Power Spectrum Coherent Integration	CPSCI
Cross-Power Spectrum Incoherent Integration	CPSII
Compensated Cross-Power Spectrum Coherent Integration	CCPSCI

**Table 2:** Averages of discrete spectra in simulation.

Algorithm	SNR = 20 dB			SNR = −10 dB		
	134 Hz	146 Hz	158 Hz	134 Hz	146 Hz	158 Hz
CPSCI/dB	−6.902	−8.037	−12.865	−9.707	−12.515	−11.155
CPSII/dB	−6.297	−8.406	−11.055	−6.262	−8.168	−11.165
CCPSCI/dB	−6.571	−6.455	−8.275	−6.331	−7.423	−9.267

**Table 3:** Averages of discrete spectra in experimental data processing.

Algorithm	199 Hz	212 Hz	225 Hz	238 Hz	251 Hz	262 Hz	282 Hz	301 Hz
CPSCI/dB	−4.378	−4.378	−6.545	−10.885	−2.201	−8.716	−6.535	−2.185
CPSII/dB	−4.374	−4.378	−6.546	−8.711	−0.032	−13.055	−4.371	−2.182
CCPSCI/dB	−0.033	−0.041	−2.222	−6.568	−0.027	−4.381	−0.035	−0.007

## Appendix A. Theory and Implementation of a Two-step Method for Time Scale Factor and Time Delay Estimations

In this appendix, a two-step method is introduced for the estimations of time scale factor and time delay. This method is proposed according to the same signal model discussed in Section 2, i.e., the received signals $r_{1}\left(n\right)$ and $r_{2}\left(n\right)$ of two hydrophones can be expressed as

(A1) $$\begin{array}{l} r_{1}\left(n\right)=s\left(n\right)+n_{1}\left(n\right)\\ r_{2}\left(n\right)=s\left(n-an-b\right)+n_{2}\left(n\right) \end{array}$$

where a is the time scale factor of $r_{2}\left(n\right)$ relative to $r_{1}\left(n\right)$, and b is the time delay between $r_{1}\left(n\right)$ and $r_{2}\left(n\right)$. $n_{1}\left(n\right)$ and $n_{2}\left(n\right)$ are the environmental noises received by the two hydrophones.

The results of the Fourier transform of $r_{1}\left(n\right)$ and $r_{2}\left(n\right)$ can be written as

(A2) $$\begin{array}{l} R_{1}\left(f\right)=S\left(f\right)+N_{1}\left(f\right)\\ R_{2}\left(f\right)=\frac{e^{-j2\pi fb}}{1-a}S\left(\frac{f}{1-a}\right)+N_{2}\left(f\right) \end{array}.$$

If $n_{1}\left(n\right)$ and $n_{2}\left(n\right)$ are the white Gaussian noises, and the sound source signal is independent from the environmental noise. Then, the cross-power spectrum $P_{12}\left(f\right)$ of $r_{1}\left(n\right)$ and $r_{2}\left(n\right)$ can be obtained from the conjugate multiplication of $R_{1}\left(f\right)$ and $R_{2}\left(f\right)$, which can be represented as

(A3) $$P_{12}\left(f\right)=\frac{e^{j2\pi fb}}{1-a}S\left(f\right)S^{*}\left(\frac{f}{1-a}\right).$$

In Equation (A3), the symbol ∗ represents the conjugate operation. If we set that

(A4) $$S\left(f\right)S^{*}\left(\frac{f}{1-a}\right)=A_{S}e^{j2\pi f\sigma },$$

where $A_{S}$ and 2πfσ are the amplitude and phase of signal power spectrum, respectively. Then, the cross-power spectrum can be rewritten as

(A5) $$P_{12}\left(f\right)=\frac{A_{S}}{1-a}e^{j2\pi f\left(b+\sigma \right)}.$$

As shown in Equation (A5), the amplitude of $P_{12}\left(f\right)$ is only under the influence of time scale factor, and the phase of $P_{12}\left(f\right)$ is affected simultaneously by the time scale factor and time delay. Therefore, the time scale factor and time delay can be estimated separately.

The first step is to estimate the time scale factor. $r_{1}\left(n\right)$ is resampled according to each value in the search range $a^{\prime }$ of time scale factor. The resampled $r_{1}\left(n\right)$ can be expressed as $r_{1}\left(n-a^{\prime }n\right)$. Then, $r_{1}\left(n-a^{\prime }n\right)$ and $r_{2}\left(n\right)$ are taken into Fourier transform to obtain $R_{1}[f/\left(1-a^{\prime }\right)]/\left(1-a^{\prime }\right)$ and $R_{2}\left(f\right)$. The amplitudes of $R_{1}[f/\left(1-a^{\prime }\right)]/\left(1-a^{\prime }\right)$ and $R_{2}\left(f\right)$ can be written as

(A6) $$\begin{array}{l} A_{1}\left(f\right)=\left|\frac{1}{1-a^{\prime }}R_{1}\left(\frac{f}{1-a^{\prime }}\right)\right|\\ A_{2}\left(f\right)=\left|R_{2}\left(f\right)\right| \end{array},$$

where |⋅| represents the modulus calculation. As a result of analysis, $A_{1}\left(f\right)$ and $A_{2}\left(f\right)$ are only affected by $a^{\prime }$. Finally, the correlation coefficients of $A_{1}\left(f\right)$ and $A_{2}\left(f\right)$ can be obtained by

(A7) $$w\left(a^{\prime }\right)=\frac{Cov\left[A_{1}\left(f\right),A_{2}\left(f\right)\right]}{\sqrt{D\left(A_{1}\left(f\right)\right)}\sqrt{D\left(A_{2}\left(f\right)\right)}},$$

where $w\left(a^{\prime }\right)$ is the correlation coefficients, Cov(⋅) and D(⋅) represent the calculations of covariance and variance, respectively. The time scale factor corresponding to the maximum of correlation coefficients is the estimation result, i.e.,

(A8) $$\bar{a}=\arg\left\{\underset{a^{\prime }}{\max}w\left(a^{\prime }\right)\right\},$$

where $\bar{a}$ is the estimation result of the time scale factor.

The second step is to estimate the time delay. ${\bar{r}}_{1}\left(n\right)$ is the resampled result of $r_{1}\left(n\right)$ according to $\bar{a}$. If $\bar{a}=a$, ${\bar{r}}_{1}\left(n\right)$ can be represented as

(A9) $${\bar{r}}_{1}\left(n\right)=r_{1}\left(n-an\right).$$

The Fourier transform of ${\bar{r}}_{1}\left(n\right)$ is

(A10) $${\bar{R}}_{1}\left(f\right)=\frac{1}{1-a}S\left(\frac{f}{1-a}\right)+\frac{1}{1-a}N_{1}\left(\frac{f}{1-a}\right).$$

Based on Equation (A2) and Equation (A10), the cross-power spectrum of ${\bar{r}}_{1}\left(n\right)$ and $r_{2}\left(n\right)$ can be expressed as

(A11) $${\bar{P}}_{12}\left(f\right)=\frac{1}{{\left(1-a\right)}^{2}}{\left|S\left(\frac{f}{1-a}\right)\right|}^{2}e^{j2\pi fb}.$$

It can be seen from Equation (A11) that the phase of ${\bar{P}}_{12}\left(f\right)$ is not affected by time scale factor anymore. Therefore, more accurate time delay can be estimated by calculating the slope of the phase of ${\bar{P}}_{12}\left(f\right)$.

## Appendix B. Parameter settings of the Methods for Spectral Estimation

### Appendix B.1. Simulated Signal Processing

The parameter settings of the methods used in simulations are described specifically as follows:

1. The lasso [33] technique is applied for performing $l_{1}\text{-}\mathrm{norm}$ minimization in the CS [34];
2. The dimension of the covariance matrix calculated by 299,000 snapshots in MUSIC [35] is 1000×1000. The number of discrete spectra to be estimated is set to three;
3. For CPSCI, CPSII, and CCPSCI, the sliding window length of LOFAR is equal to the total integration length for one incoherent or coherent integration. The number of integrations is 10, and the time length of each integration is 3 s, i.e., there is no overlap between any two integrations during each incoherent or coherent integration. The search range of time scale factor $a^{\prime }$ in CCPSCI is [−0.001,0.001] with a search step size of $5\times 10^{-5}$. The search range can be set according to the maximum speed of the moving target;
4. In order to reduce the influence from the obvious fluctuations in the continuous spectrum, the continuous spectrum of all the estimation results are made stationary with the help of detrending processing, which is achieved through polynomial fitting. The received signals are band-pass filtered in the frequency range shown in the LOFAR results before spectral estimation. Therefore, the processing of polynomial fitting is implemented in a narrowband. Generally, the continuous spectrum of sonar signal in a narrowband is linear or quadratic, which can be obtained by polynomial fitting [36,37];
5. The final spectral estimation result of each method is normalized and then displayed by LOFAR.

### Appendix B.2. Experimental Data Processing

The parameters of the methods used to process the experimental data are set as follows:

1. The lasso technique is applied for performing $l_{1}\text{-}\mathrm{norm}$ minimization in the CS;
2. The dimension of the covariance matrix calculated by 292,000 snapshots in MUSIC is 8000×8000. The number of discrete spectra to be estimated is set to eight;
3. The implementation for obtaining the incoherent and coherent integration of CPSCI, CPSII, and CCPSCI in experimental data processing is the same as in the simulation. The search range of time scale factor $\alpha^{\prime }$ in CCPSCI is [−0.0005,0.0005] with a search step size of $5\times 10^{-5}$;
4. All of the detection results are detrended by polynomial fitting to remove the fluctuating background noise;
5. The final spectral estimation result of each method is normalized and then displayed by LOFAR.

## References

[1] Ainslie M.A. (2010). Introduction. Principles of Sonar Performance Modelling.

[2] Jafri S.M.R., TBt A.P. (2014). Noise effects on surface ship passive sonar and possible ASW solution. IJTNR.

[3] Komari Alaie H., Farsi H.J. (2018). Passive sonar target detection using statistical classifier and adaptive threshold. Appl. Sci..

[4] Waite A.D., Waite A. (2002). Passive Sonar. Sonar for Practising Engineers.

[5] Lourens J. (2002). Passive sonar detection of ships with spectrograms. Proceedings of the IEEE South African Symposium on Communications and Signal Processing.

[6] Masters B.R., Madisetti V.K., Williams D.B. (1999). Signals and Systems. Handbook for Digital Signal Processing.

[7] Gröchenig K. (2001). Time-Frequency Analysis and the Uncertainty Principle. Foundations of Time-Frequency Analysis.

[8] Yan J., Sun H., Chen H., Junejo N.U.R., Cheng E. (2018). Resonance-Based Time-Frequency Manifold for Feature Extraction of Ship-Radiated Noise. Sensors.

[9] Prokopenko I., Churina A. (2008). Spectral estimation by the model of Autoregressive Moving Average and its resolution power. Proceedings of the 2008 Microwaves, Radar and Remote Sensing Symposium.

[10] Firat U., Akgül T. (2014). Spectral estimation of cavitation related narrow-band ship radiated noise based on fractional lower order statistics and multiple signal classification. Proceedings of the 2013 OCEANS-San Diego.

[11] Huang S., Sun H., Zhang H., Yu L.J. (2018). Line Spectral Estimation Based on Compressed Sensing with Deterministic Sub-Nyquist Sampling. Circuits Syst. Signal Process..

[12] Yecai G., Junwei Z., Huawei C. (2005). Special algorithm of enhancing underwater target-radiated dynamic line spectrum. J. Syst. Eng. Electron..

[13] Guo Y., Zhao J., Chen H. (2003). A novel algorithm for underwater moving-target dynamic line enhancement. Appl. Acoust..

[14] Hao Y., Chi C., Qiu L.H., Liang G.L. (2019). Sparsity-based adaptive line enhancer for passive sonars. IET Radar Sonar Navig..

[15] Yecai G., Longqing H., Yanping Z. (2007). Coherent Accumulation Algorithm Based Multilevel Switching Adaptive Line Enhancer. Proceedings of the 2007 8th International Conference on Electronic Measurement and Instruments.

[16] Farley D.T. (1995). Coherent integration. Int. Counc. Sci. Unions Middle Atmos. Program.

[17] Li X., Cui G., Yi W., Kong L.J. (2017). Sequence-reversing transform-based coherent integration for high-speed target detection. IEEE Trans. Aerosp. Electron. Syst..

[18] Rao X., Tao H., Xie J., Su J., Li W. (2015). Long-time coherent integration detection of weak manoeuvring target via integration algorithm, improved axis rotation discrete chirp-Fourier transform. IET Radar Sonar Navig..

[19] Lin L., Sun G., Cheng Z., He Z.S. (2019). Long-Time Coherent Integration for Maneuvering Target Detection Based on ITRT-MRFT. IEEE Sensors J..

[20] Wang Z., Zheng X., Chang X.L. (2019). Long-time coherent accumulation algorithm based on acceleration blind estimation. J. Eng..

[21] Yang H., Shen S., Yao X., Sheng M., Wang C. (2018). Competitive deep-belief networks for underwater acoustic target recognition. Sensors.

[22] Yang H., Li J., Shen S., Xu G. (2019). A deep convolutional neural network inspired by auditory perception for underwater acoustic target recognition. Sensors.

[23] Shen S., Yang H., Yao X., Li J., Xu G., Sheng M. (2020). Ship Type Classification by Convolutional Neural Networks with Auditory-Like Mechanisms. Sensors.

[24] Moschas F., Stiros S. (2019). Experimental evaluation of the performance of arrays of MEMS accelerometers. Mech. Syst. Signal Process..

[25] Fang E.Z., Hong L.J., Yang D.S. (2014). Self-noise analysis of the MEMS hydrophone. J. Harbin Eng. Univ..

[26] Lo K.W., Ferguson B.G. (2012). Diver detection and localization using passive sonar. Proceedings of the Acoustics.

[27] Sun T., Shan X., Chen J. (2013). Parameters estimation of LFM echoes based on relationship of time delay and Doppler shift. Proceedings of the 2012 5th International Congress on Image and Signal Processing.

[28] Ouahabi A., Kouame D. (2002). Fast techniques for time delay and Doppler estimation. Proceedings of the ICECS 2000 7th IEEE International Conference on Electronics, Circuits and Systems (Cat. No. 00EX445).

[29] Guo W., Piao S.C., Li N.S., Han X., Fu J.S. (2019). Cross-spectral time delay estimation method by Doppler estimation in frequency domain. Acta Acust..

[30] Qin L., Bingcheng Y., Wenjuan Z. (2010). Modeling of ship-radiated noise and its implement of simulator. Ship Sci. Technol..

[31] Gupta P., Kar S. (2015). MUSIC and improved MUSIC algorithm to estimate direction of arrival. Proceedings of the 2015 International Conference on Communications and Signal Processing (ICCSP).

[32] Ioannopoulos G.A., Anagnostou D., Chryssomallis M.T. (2012). A survey on the effect of small snapshots number and SNR on the efficiency of the MUSIC algorithm. Proceedings of the 2012 IEEE International Symposium on Antennas and Propagation.

[33] Tibshirani R. (1996). Regression shrinkage and selection via the lasso. J. R. Stat. Soc. Ser. B (Methodol.).

[34] Donoho D.L. (2006). Compressed sensing. IEEE Trans. Inf. Theory.

[35] Stoica P., Soderstrom T. (1991). Statistical analysis of MUSIC and subspace rotation estimates of sinusoidal frequencies. IEEE Trans. Signal Process..

[36] Müller W.G. (1996). Optimal design for local fitting. J. Stat. Plan. Inference.

[37] Abdelsalam D., Baek B.J. (2012). Curvature measurement using phase shifting in-line interferometry, single shot off-axis geometry and Zernike’s polynomial fitting. Optik.

